# Efficacy and safety of moxifloxacin in community acquired pneumonia: a prospective, multicenter, observational study (CAPRIVI)

**DOI:** 10.1186/1471-2466-14-105

**Published:** 2014-06-30

**Authors:** Ilija Kuzman, Alexandr Bezlepko, Irena Kondova Topuzovska, László Rókusz, Liudmyla Iudina, Hans-Peter Marschall, Thomas Petri

**Affiliations:** 1 University of Zagreb School of Medicine, University Hospital for Infectious Diseases “Dr. Fran Mihaljević”, Mirogojska cesta 8, 10000 Zagreb, Croatia; 2 Military Clinical Hospital N. Burdenko, Moscow, Russia; 3 University Clinic for Infectious Diseases, Medical Faculty, Skopje, Macedonia; 4 1st Department of Medicine, Military Hospital, Hungarian Defence Forces (Previously 1st Department of Medicine, National Healthcare Centre), Budapest, Hungary; 5 Pulmonology Department, National Medical Academy of Postgraduate Education, City Clinic #17, Kiev, Ukraine; 6 Bayer Vital GmbH, Leverkusen, Germany; 7 Bayer Pharma AG, Berlin, Germany

**Keywords:** Antibiotics, Pneumonia, Community acquired, CAP, Moxifloxacin

## Abstract

**Background:**

Community acquired pneumonia (CAP) is a major cause of morbidity, hospitalization, and mortality worldwide. Management of CAP for many patients requires rapid initiation of empirical antibiotic treatment, based on the spectrum of activity of available antimicrobial agents and evidence on local antibiotic resistance. Few data exist on the severity profile and treatment of hospitalized CAP patients in Eastern and Central Europe and the Middle East, in particular on use of moxifloxacin (Avelox®), which is approved in these regions.

**Methods:**

CAPRIVI (**C**ommunity **A**cquired **P**neumonia: t**R**eatment w**I**th A**V**elox® in hosp**I**talized patients) was a prospective observational study in 12 countries: Croatia, France, Hungary, Kazakhstan, Jordan, Kyrgyzstan, Lebanon, Republic of Moldova, Romania, Russia, Ukraine, and Macedonia. Patients aged >18 years were treated with moxifloxacin 400 mg daily following hospitalization with a CAP diagnosis. In addition to efficacy and safety outcomes, data were collected on patient history and disease severity measured by CRB-65 score.

**Results:**

2733 patients were enrolled. A low severity index (i.e., CRB-65 score <2) was reported in 87.5% of CAP patients assessed (n = 1847), an unexpectedly high proportion for hospitalized patients. Moxifloxacin administered for a mean of 10.0 days (range: 2.0 to 39.0 days) was highly effective: 96.7% of patients in the efficacy population (n = 2152) improved and 93.2% were cured of infection during the study. Severity of infection changed from “moderate” or “severe” in 91.8% of patients at baseline to “no infection” or “mild” in 95.5% at last visit. In the safety population (n = 2595), 127 (4.9%) patients had treatment-emergent adverse events (TEAEs) and 40 (1.54%) patients had serious TEAEs; none of these 40 patients died. The safety results were consistent with the known profile of moxifloxacin.

**Conclusions:**

The efficacy and safety profiles of moxifloxacin at the recommended dose of 400 mg daily are characterized in this large observational study of hospitalized CAP patients from Eastern and Central Europe and the Middle East. The high response rate in this study, which included patients with a range of disease severities, suggests that treatment with broader-spectrum drugs such as moxifloxacin is appropriate for patients with CAP who are managed in hospital.

**Trial registration:**

ClinicalTrials.gov identifier: NCT00987792

## Background

Community acquired pneumonia (CAP) is one of the most common infectious diseases worldwide [1]. Reported incidences of CAP in different countries range from 1.6 to 11 per 1000 adults, although precise estimates are difficult to establish, in part because no universal definition exists for diagnosing CAP [2].

CAP is a major cause of morbidity, hospitalization, mortality, and impaired quality of life, and associated with substantial societal health care burden. In Western European countries, the rate of hospitalization of patients with CAP varies between approximately 10%–60%, depending largely on the patient group under investigation [3-9]. Mortality rates of CAP range from <5% in outpatients to 10% in ward patients, and exceed 30% in patients in intensive care, illustrating the broad spectrum of severity of the disease [10]. Incidences of CAP and CAP-related mortality increase sharply with age, and are appreciably higher in men than women [9,11].

The main causative pathogens in CAP are *Streptococcus pneumoniae*, *Haemophilus influenzae*, and *Moraxella catarrhalis*, which together account for approximately 85% of cases [12]. Additional “atypical” pathogens in CAP include *Mycoplasma pneumoniae*, *Chlamydophila pneumoniae*, and *Legionella pneumophila.* Many CAP patients have mixed infections including both typical and atypical pathogens [13].

Instant diagnosis of the causative organism would be optimal for managing CAP; however, the responsible pathogen is usually not known at the time of CAP diagnosis and no pathogen is identified in up to 50% of patients even after extensive testing for several days [14]. Since rapid initiation of antibiotic treatment of CAP is mandatory, an empirical antibiotic therapy is required taking into consideration the spectrum of efficacy of available agents and local evidence on antimicrobial resistance [15]. Guidelines on empirical treatment from the Infectious Disease Society of America (IDSA)/American Thoracic Society (ATS) recommend an antipneumococcal fluoroquinolone (e.g., moxifloxacin or levofloxacin) or a combination of β-lactam and macrolide for CAP patients hospitalized on a general ward [15]. The guidelines of the European Respiratory Society also recommend moxifloxacin or levofloxacin as a treatment option for empirical therapy for hospitalized CAP patients with or without the need of intensive care [16].

Moxifloxacin is a fourth-generation fluoroquinolone with a broad spectrum of activity against microorganisms isolated in CAP, including multi-resistant pneumococci and pathogens such as *M. catarrhalis* and *H. influenzae* with resistance to penicillins, macrolides, and tetracyclines. Moxifloxacin also possesses activity against atypical pathogens including *L. pneumophila*, *C. pneumoniae,* and *M. pneumoniae*[17-20].

Moxifloxacin at the recommended dose of 400 mg once daily has been investigated in prospective, randomized, double-blind clinical trials and meta-analyses in patients with mild, moderate, and severe CAP in community- and hospital-based settings (e.g., [21-32]).

Moxifloxacin is generally very well tolerated by patients, with low incidences of adverse events in clinical and post-marketing studies [33]. A meta-analysis that included 14 randomized controlled trials, consisting of 6923 patients, confirmed that moxifloxacin is as effective and well tolerated as other recommended antibiotics in treating CAP and has a pathogen eradication rate superior to β-lactam-based therapy [32]. Studies in CAP patients also show that moxifloxacin reduces length of hospital stay compared with standard therapies, with potential significant cost savings [34,35].

Most moxifloxacin studies in CAP have been randomized trials, but observational studies also provide valuable information relevant to practice [36].

The primary objective of the current observational study – the CAPRIVI (Community Acquired Pneumonia: tReatment wIth AVelox® in hospItalized patients) study – was to evaluate the distribution of CRB-65 severity index at baseline in patients hospitalized with CAP. The CRB-65 is a validated tool for risk evaluation that can be used for rapid selection of treatment strategies in CAP. The reliability of the CRB-65 has been confirmed in more than 5000 patients in Western Europe and Asia, mostly in hospitalized patients or patients seen initially in emergency departments [37-42]. The distribution of CRB-65 scores has not previously been reported in hospitalized CAP patients from South Eastern Europe, the Community of Independent States, or the Middle East.

The majority of moxifloxacin studies have been conducted in Western Europe or Northern America, where intravenous moxifloxacin has been available for a number of years. Secondary objectives of the CAPRIVI study were to collect data on the efficacy and safety of moxifloxacin in CAP from South Eastern Europe, the Community of Independent States, or the Middle East, where data from routine practice have not previously been collected. Patients from France were included in the CAPRIVI study, since intravenous moxifloxacin has only recently become available in this country.

## Methods

### Study design

The CAPRIVI study was a prospective, multicenter, observational study conducted at 247 investigational centers in 12 countries: Croatia, France, Hungary, Kazakhstan, Jordan, Kyrgyzstan, Lebanon, Republic of Moldova, Romania, Russia, Ukraine, and Macedonia, between September 15, 2009 and June 20, 2011.

The study consisted of an assessment at baseline and an observational period between the initiation and completion of treatment with moxifloxacin (Avelox®) in patients hospitalized with CAP. One, or at most two, follow-up study visits were planned for each patient after the initial assessment.

### Patients and investigators

Male or female patients aged 18 years and above who were hospitalized with a diagnosis of CAP were included in the study after the decision to prescribe moxifloxacin was made by treating physicians. The decision to prescribe moxifloxacin was made by physicians based on their medical judgment and in accordance with the locally available Summary of Product Characteristics for moxifloxacin. The diagnosis of CAP and any concomitant diseases was based on local medical practice.

The majority of patients (85.5%, n = 1840/2152) entered hospital on the first day of moxifloxacin therapy; 45.2% of patients were hospitalized as emergency cases and 43.6% were referred to hospital. Patients were most frequently treated in the pneumology unit (54.3%), followed by internal medicine (20.0%) and infectious disease units (10.3%). No information on the treating unit was given in the remaining cases.

Study exclusion criteria were limited to the contraindications to the use of moxifloxacin as described in the local product information. Use of a concomitant anti-infective treatment was an additional exclusion criterion. Data on disease characteristics and risk factors were collected from patients before the initiation of moxifloxacin treatment.

The study protocol was approved by the local independent ethics committee or institutional review board, as applicable in each country. Committee and review board approvals were provided as follows: Croatia: the National Ethics Committee (EC); France: the EC of the National Council of Physicians; Hungary: the National Ethics Committee; Kazakhstan: the EC of the National Center of Expertise of Medicinal Drugs, Medical Equipment and Items of Medical Utility; Kyrgyzstan: the Department of Drug Provision and Medical Equipment under the Ministry of Health of the Kyrgyz Republic; Macedonia: the EC for Medical Trials from the Medical Faculty in Skopje as well by the Commission for Clinical Trials of the National Drug Agency; Republic of Moldova: the Ministry of Health; Romania: the National Agency for Medicines and Medical Devices and the National EC for the Clinical Study of Medicines; Russia: the National Intercollegiate EC; and Ukraine: the Central Ethics Committee of Ministry of Health. No EC approval was required in the Lebanon and Jordan at the time this study was conducted. All patients provided written, informed consent at the start of the study, in accordance with and as required by local regulations.

### Centralized risk-based study monitoring and quality review

Data fabrication is a rare form of scientific misconduct in clinical trials, but when it does occur it has serious consequences [43]. Non-interventional trials may be even more prone to data fabrication, since quality assurance has only recently been introduced for observational studies [44]. Data fabrication was considered to be the highest risk to the data quality of this study. This is one of the first non-interventional studies where quality assurance procedures (described below) were used to detect data falsification.

The US Food and Drug Administration (FDA) recently published recommendations on centralized monitoring practices, which should improve a sponsor’s ability to ensure the quality of trial data [45]. It is surprisingly hard to fabricate data without leaving all kinds of statistical clues that can be detected from the data [46].

A set of central monitoring and biostatistical testing procedures tailored to the study protocol were implemented to identify investigational centers suspicious for fraudulent activity. Tests were implemented, for example, to detect a very large number of recruited patients, multiple patients enrolled on the same day, multiple patients with the same pattern of follow-up visits, a short duration of recruitment in relation to the number of the recruited patients, low numbers of queries and low numbers of missing data per patient, low numbers of adverse events per patient, low numbers of patients lost to follow up, and obvious similarities in laboratory values. These tests compared the site performance relative to all other sites. Points were given per test depending on the quartile that the site fell into and whether the upper or lower quartile was suspicious for fraudulent activity. These points were summed to calculate the fraud score for each site. The higher the score, the higher was the risk that falsified data were submitted by the site.

Focusing on centers with a high fraud score, telephone interviews were conducted with a set of predefined questions to decide whether a suspicion of fraud could or could not be rejected. The interviews were carefully documented and the results were evaluated by the interviewers and study managers. All patients from sites where the suspicion of fraudulent activity could not be rejected during the telephone interview were excluded from analysis.

### Study medication

Moxifloxacin contains a C-8 methoxy substitute that increases bactericidal activity, decreases the risk of selecting resistant mutants, and enhances activity against Gram-positive bacteria [47]. Moxifloxacin has beneficial pharmacokinetic and pharmacodynamic properties, is strongly targeted to alveolar tissue, and demonstrates rapid initial killing with high eradication rates [48].

Moxifloxacin was prescribed in accordance with guidelines from the European Medicines Agency, the FDA, and local regulations (e.g., AVELOX® [moxifloxacin hydrochloride] Summary of Product Characteristics) [49,50].

The dose of moxifloxacin recommended for treatment of CAP in the study was 400 mg once every 24 hours, consistent with the local Summary of Product Characteristics. Moxifloxacin could be administered exclusively as intravenous therapy or as sequential therapy consisting of intravenous followed by oral administration. The method of administration of moxifloxacin was selected by the treating physician. The recommended duration of moxifloxacin therapy for CAP in guidelines is 7 to 14 days, depending on the severity of disease and clinical response. Final decisions on the dose and duration of moxifloxacin therapy and on the use of concomitant medications were made by the attending physician.

### Efficacy and safety assessments

The primary study objective was assessment of the distribution of CRB-65 score at baseline. The CRB-65 score was calculated by awarding one point each for the following: presence of mental confusion, respiratory rate ≥30 breaths/min, systolic blood pressure <90 mmHg or diastolic blood pressure ≤60 mmHg, and aged ≥65 years [5,37,51-53]. The mortality rate in patients with a CRB-65 score of 0 is below 1%, with a score of 1–2 about 5% and with a score of 3 or 4 up to 25%. Patients with a CRB-65 score of 3 or 4 should be considered for urgent hospital admission [40].

Additional efficacy assessments, performed at the initial visit and the last follow-up visit after moxifloxacin therapy, included standard diagnostic measures in daily practice: chest radiography, blood laboratory tests, core body temperature, and microbiologic tests; clinical signs and symptoms including dyspnea, cough, sputum character, and thoracic pain (classified as “relieved”, “improved”, “unchanged” or “worsened” vs baseline); and the overall condition of the patient and overall severity of infection as judged by the physician. Time to improvement, rate of cure (i.e., a return to the status prior to onset of CAP), time to hospital discharge, physicians’ rating of symptom improvement, and patients’ satisfaction with moxifloxacin therapy were also assessed.

Safety and tolerability were assessed by treatment-emergent adverse events (TEAEs) reported during the study, coded using the Medical Dictionary for Regulatory Activities (MedDRA) version 15.0, and categorized by seriousness and relationship to treatment, the frequency of premature discontinuations of moxifloxacin therapy, referral rates, and hospitalizations due to failure of therapy.

### Statistical analyses

Efficacy and safety analyses were exploratory and observational, as appropriate for non-interventional studies. Categorical and quantitative (continuous) data were analyzed by descriptive statistics including means, standard deviations (SDs), and minimum and maximum values. Non-missing data are presented throughout.

The efficacy population included all patients who took at least one dose of study medication and provided information on the efficacy of treatment. The safety population included all patients who received at least one dose of study medication and provided information on adverse events.

According to sample size calculations, 2655 patients were required to be included for analysis of the main study objectives.

## Results

### Centralized risk-based study monitoring and quality review

From a total of 253 sites, the 14 centers with the highest fraud scores were selected for telephone interviews. Two of these 14 centers were excluded because they were unwilling to be interviewed by telephone. For two other centers, the suspicion of fraud could not be rejected from the answers given during the telephone interview, e.g., because the patient files were not available to provide answers to prespecified questions. These four centers, with a total of 135 patients, were therefore excluded from data analysis. In the remaining 10 centers, plausible answers were received and the source data were available for review. Thus the suspicion of fraud was rejected for these latter centers and their data were included in the analyses.

### Patient population

**Figure 1:**
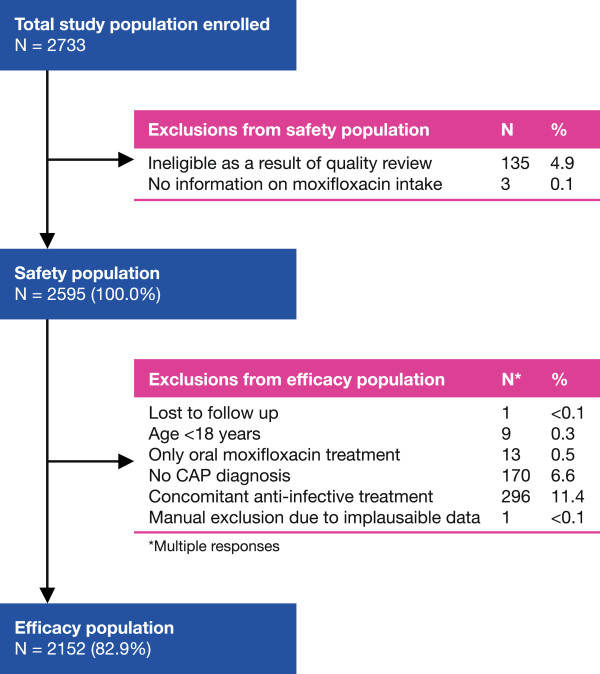
Patient disposition.

A total of 2733 patients were enrolled in the study. Of these, 135 patients (as mentioned above) were excluded because their recruiting site failed to pass quality review. Three additional patients were excluded because data were not available on moxifloxacin intake, leaving 2595 patients for inclusion in the safety population. Of these 2595 patients, 443 were excluded from the efficacy population, in the majority of cases (n = 296) because of concomitant other anti-infective treatment, leaving 2152 patients evaluable for efficacy (Figure 1).

Demographic and disease characteristics of the efficacy population are described in Table 1. Patient ages ranged from 18 to 100 years (mean 53.3 ± 17.9 years), with 29.2% of patients aged above 65 years. More men (56.7%) than women participated in the study, while White patients constituted the majority (85.2%). Over one third of patients were documented as past (12.8%) or current (23.3%) smokers.

**Table 1:** Patient demographics and disease characteristics (efficacy population)

**Parameter**	**Total N = 2152 (100%)**
Gender, n (%)	
Male	1220 (56.7)
Female	918 (42.7)
Missing	14 (0.7)
Mean (SD) age, y (n = 2136)	53.3 (17.9)
Mean (SD) weight, kg (n = 2117)	77.3 (15.0)
Mean (SD) height, cm (n = 2099)	170.9 (9.0)
Mean (SD) BMI, kg/m^2^ (n = 2098)	26.5 (4.8)
Race, n (%)	
White	1833 (85.2)
Asian	119 (5.5)
Black	3 (0.1)
Other	4 (0.2)
Missing	193 (9.0)
Smoking status, n (%)	
Non-smoker	1331 (61.8)
Present smoker	501 (23.3)
Past smoker	276 (12.8)
Missing	44 (2.0)
Type of vaccination, n (%)	
None	1546 (71.8)
Pneumococcus	8 (0.4)
Influenza	237 (11.0)
Both	18 (0.8)
Missing	343 (15.9)
Pneumonia episodes in past 12 months	
Yes	187 (8.7)
No	1838 (85.4)
Unknown	81 (3.8)
Missing	46 (2.1)
Hospitalization in past 12 months	
Yes	319 (14.8)
Due to CAP	112 (35.1)
Other	178 (55.8)
Missing	40 (12.5)
No	1740 (80.9)
Unknown	53 (2.5)
Missing	40 (1.9)

The majority of patients (71.8%) had received no vaccination, neither against pneumococcus nor against influenza. A pneumonia episode in the previous 12 months was reported by 8.7% of patients. Among the patients hospitalized in the last 12 months (14.8% of the efficacy population), 35.1% named CAP as the reason for hospitalization (Table 1).

All patients were hospitalized in the current study because of a diagnosis of CAP, and no patients were hospitalized for concomitant disease. One or more concomitant diseases were reported by 70.4% of patients. A risk factor for CAP or a prespecified concomitant disease of special interest was recorded in 56.5% of patients, including: chronic obstructive pulmonary disease (31.7% of patients), cardiac ischemia (18.1%), diabetes mellitus (9.7%), congestive heart failure (8.4%), and asthma (7.5%). One or more concomitant medications were taken by 79.5% of patients. As expected, the majority of these patients (71.2%) received concomitant medication for the respiratory system. The mucolytic ambroxol/ambroxol hydrochloride was the most frequently used medication (27.9% of all patients), followed by paracetamol (14.2%), acetylcysteine (11.6%), and furosemide (10.1%).

A previous antibiotic had been administered to 41.4% of patients within 14 days before starting moxifloxacin therapy, most commonly amoxicillin (with or without clavulanic acid), ceftriaxone, or azithromycin. Patients were switched to moxifloxacin for the current CAP episode most frequently because of a lack of efficacy with the previous antibiotic (87.3%).

Patients experienced CAP symptoms for a mean (SD) of 5.0 ± 3.8 days (range, 0.0–35.0 days) before the start of moxifloxacin therapy. The majority of patients (85.5%) started moxifloxacin on the day of hospitalization (Table 2). Most patients were hospitalized as emergency cases (45.2%) or referred by physicians (43.6%), with another 7.2% of patients self-referred and 2.9% already hospitalized.

**Table 2:** Duration between day of hospitalization and start of moxifloxacin therapy (efficacy population)

**Duration (days)**	**Total N = 2152 (100.0%)**	
	**n**	**(%)**
0	1840	(85.5)
1	107	(5.0)
2	55	(2.6)
3	46	(2.1)
4	32	(1.5)
5	36	(1.7)
6	34	(1.1)
Missing	12	(0.6)

### Baseline assessments

#### CRB-65 score

**Figure 2:**
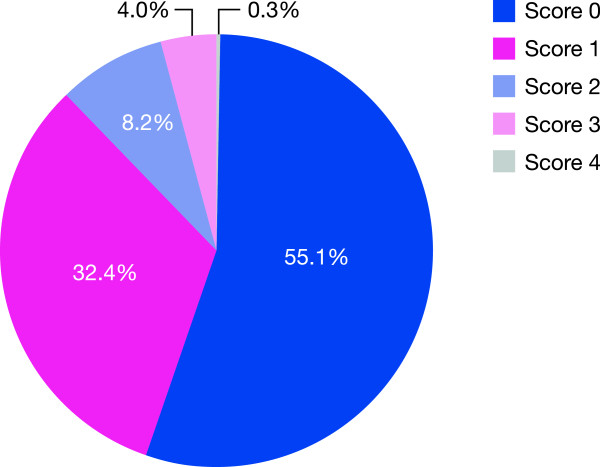
Distribution of CRB-65 scores in study population at baseline.

Distributions of CRB-65 scores were as follows: 55.1% of patients scored 0, 32.4% scored 1, 8.2% scored 2, 4.0% scored 3, and 0.3% scored 4 (Figure 2). On categorizing patients into groups based on low or high severity index (i.e., CRB-65 score <2 vs ≥2), 87.5% of patients (95% confidence interval [CI], 86.0–89.0) had a low index and 12.5% (95% CI, 11.0–14.0) had a high index.

Considering the individual CRB-65 score components, mental confusion was present in 8.6% of patients, respiratory rate ≥30 breaths/min in 7.2%, systolic blood pressure <90 mmHg or diastolic blood pressure ≤60 mmHg in 14.0%, and age ≥65 years in 29.2% of patients.

#### Diagnostic measures

Chest radiography was performed at the initial visit in 98.9% of patients, showing at least one of the following: unilateral infiltrate in 76.0% of patients, bilateral infiltrate in 19.8%, pleural effusion in 6.7%, and multilobar involvement in 4.3%. A normal lung appearance was observed in 0.7%. In these latter cases as well as in those with a CAP diagnosis without a confirmatory chest radiograph, CAP was diagnosed by other procedures, according to the physicians’ experience. Mean (SD) laboratory test results in patients with available data were: C-reactive protein: 109.6 ± 267.1 mg/L (n = 766), arterial blood partial pressure of oxygen: 66.6 ± 14.3 mmHg (n = 484), blood pH: 7.41 ± 0.07 (n = 774), and oxygen saturation: 91.9 ± 5.7 (n = 1459). The mean (SD) value for white blood count was 11.6 ± 5.8 giga/L (n = 2120); 67 (3.1%) of patients had a WBC count of <4 giga/L (3.1%) and 1054 (49.0%) had a WBC count of >11 giga/L.

Microorganisms were isolated/diagnosed in 507 (23.6%) patients. Methods for the isolation/diagnosis of microorganisms included (use of more than one method was possible): culture of respiratory secretions (48.0% of cases), blood culture (16.5%), blood serology (9.2%), or urinary antigen (2.6%). The method was not recorded for 42.8% of patients who had microorganisms isolated/diagnosed.

The microorganisms isolated/diagnosed included *S. pneumoniae* in 10.1% of patients, *H, influenzae* in 4.2%, *Staphylococcus aureus* in 2.9%, *C. pneumoniae* in 1.8%, *M. pneumoniae* in 1.5%, *L. pneumophila* in 1.1%, *M. catarrhalis* in 0.9%, and Enterobacteriaceae/“Other” in 5.2%. Rates of phenotypic resistance in the microorganisms are described in Table 3.

**Table 3:** **Phenotypic resistance of isolated microorganisms (efficacy population**)

**Microorganism**	**Resistance status**			
***Streptococcus pneumoniae***	Penicillin-R*	Penicillin-I*	Penicillin-S*	**Not available**
**n = 217 (100%)**	27/217 (12.4%)	2/217 (2.3%)	69/217 (31.8%)	116/217 (53.5%)
***Haemophilus influenzae***	**β-lactamase pos.**	---	**β-lactamase neg.**	**Not available**
**n = 90 (100%)**	6/90 (6.7%)		20/90 (22.2%)	64/90 (71.3%)
***Moraxella catarrhalis***	**β-lactamase pos.**	---	**β-lactamase neg.**	**Not available**
**n = 19 (100%)**	2/19 (10.5%)		3/19 (15.8%)	15/19 (73.7%)
***Staphylococcus aureus***	**MRSA**	---	**MSSA**	**Not available**
**n = 62 (100%)**	2/62 (3.2%)		17/62 (21.4%)	41/62 (66.1%)
	**Oxacillin-R**		**Oxacillin-S**	
	0/62 (0%)		2/62 (3.2%)	
**Enterobacteriaceae spp.**	**ESBL producer**	---	**Non-ESBL producer**	**Not available**
**n = 211 (100%)**	2/111 (1.8%)		11/111 (9.9%)	98/111 (88.3%)

*R, I, S = resistant, intermediate resistant, sensitive, respectively. ESBL, extended-spectrum β-lactamase; MRSA, methicillin-resistant *Staphylococcus aureus*; MSSA, methicillin-sensitive *Staphylococcus aureus*.

### Moxifloxacin treatment

The majority of patients treated with moxifloxacin (83.7%) received sequential intravenous followed by oral administration, and the remainder received exclusively intravenous treatment. In total, 97.1% of patients in the sequential treatment group and 99.7% in the intravenous treatment group received the recommended daily dose of 400 mg moxifloxacin. No information on the daily dose was given for 2.9% and 0.3% of patients in the respective groups. The mean duration of moxifloxacin treatment until symptom improvement or treatment discontinuation was 10.0 ± 3.0 days (range, 2.0–39.0 days) in the combined group, consisting of 10.5 ± 2.7 days in the sequential treatment group (mean 4.1 days for intravenous and 6.5 days for oral administration) and 7.3 ± 3.2 days in the intravenous treatment group.

#### Assessments during moxifloxacin treatment

In total, 84.8% of patients had two follow-up visits. The mean (SD) duration between the start of moxifloxacin therapy and the first and second follow-up visits was 4.7 ± 3.6 days and 11.0 ± 5.2 days, respectively. The last follow-up visit was a mean of 10.6 ± 5.5 days after the initiation of moxifloxacin therapy.

#### Course of signs and symptoms

The overall condition of patients changed from primarily “serious” or “critical” (68.1% of patients) at the initial visit to mainly “good” or “fair” (97.6%) at the last visit (Table 4). The severity of infection changed from “moderate” or “severe” in the majority of patients (91.8%) to “no infection” or “mild” in most (95.5%) at the last visit. Improvements in the severity of dyspnea, cough, sputum character, and thoracic pain and in rates of abnormal auscultation, core temperature, and chest radiography are shown in Table 4.

**Table 4:** Investigations at initial and follow-up visits (efficacy population)

**Investigations**	**Visits**							
	**Initial**		**1st follow-up**		**2nd follow-up**		**Last**	
	**n**	**(%)**	**n**	**(%)**	**n**	**(%)**	**n**	**(%)**
Patient’s condition								
Total patients	2152	(100.0)	2139	(100.0)	1824	(100.0)	2139	(100.0)
Missing	2	(0.1)	5	(0.2)	7	(0.4)	9	(0.4)
Good	65	(3.0)	713	(33.3)	1349	(74.0)	1549	(72.4)
Fair	619	(28.8)	1230	(57.5)	439	(24.1)	539	(25.2)
Serious	1385	(64.4)	176	(8.2)	21	(1.2)	31	(1.4)
Critical	81	(3.8)	15	(0.7)	8	(0.4)	11	(0.5)
Severity of infection								
Total patients	2152	(100.0)	2139	(100.0)	1824	(100.0)	2139	(100.0)
Missing	2	(0.1)	5	(0.2)	14	(0.8)	15	(0.7)
No infection	0	(-)	339	(15.8)	1482	(81.3)	1670	(78.1)
Mild	174	(8.1)	1174	(54.9)	277	(15.2)	373	(17.4)
Moderate	1378	(64.0)	565	(26.4)	32	(1.8)	54	(2.5)
Severe	598	(27.8)	56	(2.6)	19	(1.0)	27	(1.3)
Dyspnea								
Total patients	2152	(100.0)	2139	(100.0)	1824	(100.0)	2139	(100.0)
Missing	7	(0.3)	13	(0.6)	12	(0.7)	15	(0.7)
None	283	(13.2)	932	(43.6)	1488	(81.6)	1679	(78.5)
Mild	546	(25.4)	896	(41.9)	279	(15.3)	381	(17.8)
Moderate	959	(44.6)	269	(12.6)	32	(1.8)	46	(2.2)
Severe	357	(16.6)	29	(1.4)	13	(0.7)	18	(0.8)
Cough								
Total patients	2152	(100.0)	2139	(100.0)	1824	(100.0)	2139	(100.0)
Missing	6	(0.3)	7	(0.3)	11	(0.6)	11	(0.5)
None	49	(2.3)	335	(15.7)	1067	(58.5)	1177	(55.0)
Mild	321	(14.9)	1115	(52.1)	672	(36.8)	850	(39.7)
Moderate	1142	(53.1)	638	(29.8)	70	(3.8)	93	(4.3)
Severe	634	(29.5)	44	(2.1)	4	(0.2)	8	(0.4)
Sputum character								
Total patients	2152	(100.0)	2139	(100.0)	1824	(100.0)	2139	(100.0)
Missing	4	(0.2)	8	(0.4)	12	(0.7)	13	(0.6)
None	338	(15.7)	582	(27.2)	1202	(65.9)	1344	(62.8)
Clear	151	(7.0)	713	(33.3)	489	(26.8)	602	(28.1)
Mucoid	783	(36.4)	713	(33.3)	110	(6.0)	161	(7.5)
Purulent	876	(40.7)	123	(5.8)	11	(0.6)	19	(0.9)
Thoracic pain								
Total patients	2152	(100.0)	2139	(100.0)	1824	(100.0)	2139	(100.0)
Missing	11	(0.5)	18	(0.8)	15	(0.8)	18	(0.8)
None	696	(32.3)	1416	(66.2)	1686	(92.4)	1918	(89.7)
Mild	554	(25.7)	539	(25.2)	98	(5.4)	166	(7.8)
Moderate	775	(36.0)	154	(7.2)	21	(1.2)	31	(1.4)
Severe	116	(5.4)	12	(0.6)	4	(0.2)	6	(0.3)
Auscultation								
Total patients	2152	(100.0)	2139	(100.0)	1824	(100.0)	2139	(100.0)
Missing	27	(1.3)	74	(3.5)	48	(2.6)	63	(2.9)
Normal	84	(3.9)	794	(37.1)	1591	(87.2)	1821	(85.1)
Pathological	2041	(94.8)	1271	(59.4)	185	(10.1)	255	(11.9)
Core temperature*								
Total patients	2152	(100.0)	2139	(100.0)	1824	(100.0)	2139	(100.0)
Missing	30	(1.4)	93	(4.3)	156	(8.6)	187	(8.7)
Low	0	(-)	1	(<0.1)	0	(-)	0	(-)
Normal	77	(3.6)	552	(25.8)	628	(34.4)	724	(33.8)
Mild	147	(6.8)	848	(39.6)	936	(51.3)	1052	(49.2)
Moderate	531	(24.7)	585	(27.3)	93	(5.1)	159	(7.4)
Severe	1367	(63.5)	60	(2.8)	11	(0.6)	17	(0.8)
Chest radiography**								
Total patients	2152	(100.0)	2139	(100.0)	1824	(100.0)	2139	(100.0)
Missing	14	(0.7)	51	(2.4)	16	(0.9)	31	(1.4)
Not done	8	(0.4)	1230	(57.5)	397	(21.8)	483	(22.6)
Normal	16	(0.7)	292	(13.7)	1178	(64.6)	1299	(60.7)
Pleural effusion	144	(6.7)	41	(1.9)	24	(1.3)	27	(1.3)
Unilateral infiltrate	1635	(76.0)	425	(19.9)	178	(9.8)	263	(12.3)
Bilateral infiltrate	427	(19.8)	114	(5.3)	34	(1.9)	41	(1.9)
Multilobar involvement	92	(4.3)	18	(0.8)	11	(0.6)	12	(0.6)

*Comparison of temperature ranges: Low: <36.0°C, Normal: 36.0°C – <37.5°C, Mild: 37.5°C – <38.0°C, Moderate: 38.0°C – <39.0°C, Severe: ≥39.0°C; **Multiple responses.

Symptoms were rated as “relieved” or “improved” over the course of therapy in the majority of patients for dyspnea (95.3%), cough (93.3%), sputum character (94.7%), thoracic pain (95.8%), and auscultation (84.2%) (Table 5). Symptoms were unchanged in a small number of patients (ranging from 2.1% for thoracic pain to 11.8% for auscultation) and worsening of symptoms was reported in a very small proportion (ranging from 0.1% for auscultation to 1.3% for temperature).

**Table 5:** Course of clinical signs and symptoms from initial to last follow up visit (efficacy population)

**Course of symptoms**	**Dyspnea**		**Cough**		**Sputum character**		**Thoracic pain**		**Auscultation**		Temperature*	
	**n**	**(%)**	**n**	**(%)**	**n**	**(%)**	**n**	**(%)**	**n**	**(%)**	**n**	**(%)**
Total	2152	(100.0)	2152	(100.0)	2152	(100.0)	2152	(100.0)	2152	(100.0)	2152	(100.0)
Relieved	1677	(77.9)	1174	(54.6)	1343	(62.4)	1914	(88.9)	1811	(84.2)	722	(33.6)
Improved	374	(17.4)	832	(38.7)	695	(32.3)	148	(6.9)	0	(-)	1101	(51.2)
Unchanged	61	(2.8)	104	(4.8)	65	(3.0)	45	(2.1)	253	(11.8)	98	(4.6)
Worsened	9	(0.4)	13	(0.6)	21	(1.0)	10	(0.5)	2	(0.1)	28	(1.3)
Missing	31	(1.4)	29	(1.3)	28	(1.3)	35	(1.6)	86	(4.0)	203	(9.4)

*Comparison of temperature ranges: Low: <36.0°C, Normal: 36.0°C – <37.5°C, Mild: 37.5°C – <38.0°C, Moderate: 38.0°C – <39.0°C, Severe: ≥39.0°C.

The mean core temperature was 39.2°C ± 0.9°C (range: 36.0°C to 42.0°C) at baseline visit and 37.5°C ± 0.5°C (range: 36.0°C to 40.9°C) at the last visit. A return to normal temperature (i.e., ≤37.5°C) occurred at a mean of 2.8 ± 1.5 days (Table 6). Temperature was rated as “relieved” or “improved” in the majority of patients (84.8%) from the initial to the last visit.

**Table 6:** Duration until return to normal temperature (efficacy population)

**Duration until ≤37.5°C (days)**	**Total N = 2152 (100%)**			
	n*	**(%)**	**n cum**	**(%) cum**
1	273	(14.4)**	273	(12.7)
2	656	(34.6)**	929	(43.2)
3	557	(29.4)**	1486	(69.1)
4	214	(11.3)**	1700	(79.0)
5	116	(6.1)**	1816	(84.4)
6	30	(1.6)**	1846	(85.8)
7	27	(1.4)**	1873	(87.0)
8	7	(0.4)**	1880	(87.4)
9	5	(0.3)**	1885	(87.6)
10	8	(0.4)**	1893	(88.0)
11	2	(0.1)**	1895	(88.1)
20	1	(0.1)**	1896	(88.1)
No return to normal T	37	(1.7)	1933	(89.8)
Patient not febrile at start	189	(8.8)		
T not taken continuously	12	(0.6)		
Missing	19	(0.9)		
Total	2152	(100.0)	2152	(100.0)

*Multiple responses; **Percentage of total return to normal temperature (n = 1896).

T, temperature.

#### Time to improvement and cure rates

**Figure 3:**
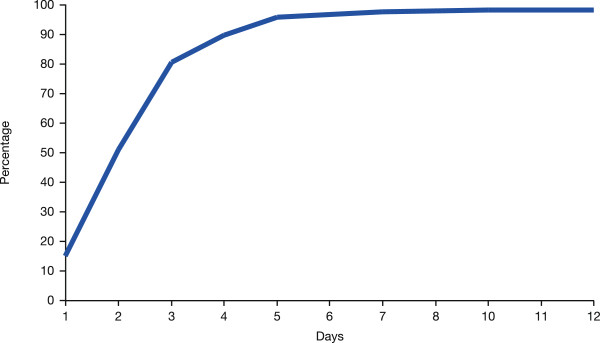
Cumulative increase in proportion of patients who showed improvement (efficacy population).

In total, 96.7% of patients in the efficacy population were reported to improve, i.e., feel better, during the study, while only 2.8% did not improve (Figure 3). Improvement was achieved in 78.9% of patients after 3 days, 88.3% after 4 days, and 94.4% after 5 days. The mean duration until improvement was 2.7 ± 1.3 days.

Cure of infection was reported in 93.2% of patients at the end of therapy, versus 5.2% who were not cured (data were missing in 1.6% of patients). Stratified analyses by symptoms, diagnostic measures, vital signs, and previous antibiotic use at baseline identified no substantial differences in the rate of cure of CAP between subgroups. Cure rates in patients with mild, moderate, or severe infection, for example, were 94.3%, 94.6%, and 89.6%, respectively.

#### Time to hospital discharge

A total of 93.2% of patients were discharged from hospital after a mean of 10.4 ± 5.9 days of moxifloxacin therapy. Stratified analysis showed that duration of hospitalization was longer in patients with a higher CRB-65 score at baseline (mean 13.5 and 10.4 days for scores of ≥2 and <2, respectively) and in patients who did not versus who did achieve a cure by moxifloxacin (mean 15.5 and 10.2 days). The most frequently identified reasons why patients were not discharged from hospital were concomitant diseases (0.6%) and failure of moxifloxacin therapy (0.3%).

#### Safety assessments

Moxifloxacin was well tolerated, with low rates of TEAEs and serious TEAEs. Between the initial visit and the last follow-up visit, there were 171 TEAEs documented in 127 (4.9%) of the 2595 patients in the safety population. In the judgment of investigators, 109/171 TEAEs (63.7%; in 87 patients) were related to moxifloxacin treatment. Drug-related TEAEs (in ≥0.10% of the safety population) included diarrhea (1.23% of patients), nausea (0.50%), urticaria (0.19%), dizziness (0.15%), dysgeusia (0.15%), and headache (0.12%). No hepatic side effects and no serious skin lesions were reported.

In total, 110/171 TEAEs (in 87 patients) resolved during the study, two TEAEs (one patient) resolved with sequelae, 12 TEAEs (10 patients) were resolving, and four TEAEs (three patients) were unresolved. TEAEs were rated serious on 62 occasions (40 patients), based on classifications including “necessary or prolonged hospitalization” (31 patients), “fatal outcome” (31 patients), “important medical event” (four patients), “life-threatening event” (four patients), and “disability/incapacity” (one patient). Of the 62 serious events, 11 were classified as drug-related (seven patients). Twenty-seven TEAEs (19 patients) were fatal; none of the fatal TEAEs were considered drug-related.

No change in moxifloxacin dose was required for 72/171 (42.1%) TEAEs; moxifloxacin was completely withdrawn for 36 TEAEs (21.2%) and was interrupted (with subsequent resumption of moxifloxacin after TEAEs had ceased) for 31 TEAEs (18.1%). Moxifloxacin therapy was prematurely discontinued in 3.3% of patients (n = 72), mainly owing to adverse events (n = 34), lack of efficacy (n = 24), and bacterial resistance (n = 6). Another antibiotic for treatment of CAP was initiated in 5.5% of patients (n = 119) following moxifloxacin.

#### Physicians’ rating of symptom improvement and patients’ satisfaction with moxifloxacin therapy

**Figure 4:**
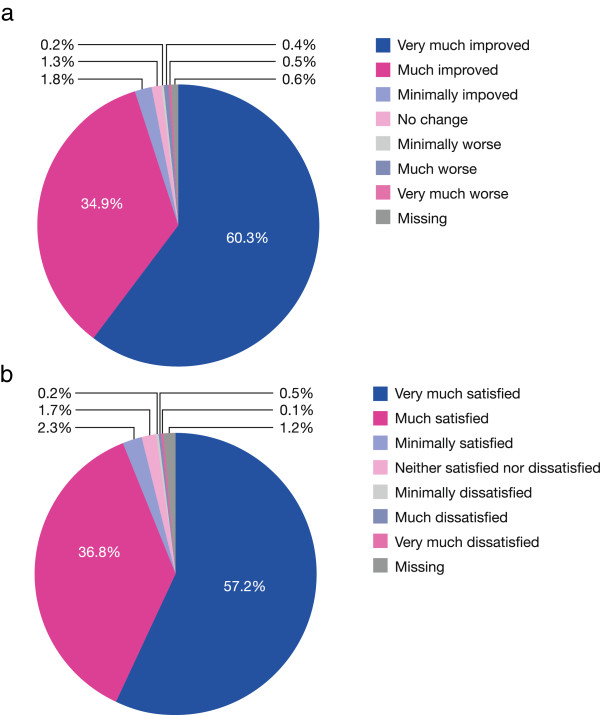
**Assessments of improvement and satisfaction. (a)** Physicians’ rating of improvement during moxifloxacin therapy; **(b)** patients’ satisfaction with moxifloxacin therapy (efficacy population).

Physicians rated the symptoms of CAP as either “very much improved” or “much improved” in 95.3% of patients (Figure 4a). Minimal improvement, or no change in symptoms, was recorded in 3.1%, and a worsening of symptoms in 1.0% of patients. Stratified analysis showed that the symptoms of CAP were improved less in patients with increasing age or coexisting risk factors.Physicians reported that 94.1% of patients were “very much satisfied” or “much satisfied” with moxifloxacin therapy (Figure 4b). Minimal satisfaction, or indifference to therapy, was reported by 3.9% and dissatisfaction by 0.8% of patients.

## Discussion

This non-interventional, naturalistic, observational study evaluated the baseline CRB-65 status and the efficacy and safety of moxifloxacin treatment in 2733 enrolled patients hospitalized with a current episode of CAP. A notable feature of this study is the well-documented patient history including previous episodes of CAP, vaccination history, concomitant diseases, and co-medications. Another notable feature in this observational study is the use of quality assurance measures to confirm the validity of the data analyzed, leading to the exclusion of approximately 5% of the originally included patients since the suspicion of data falsification by the investigators could not be excluded. Although this led to a reduction in the overall number of the patients, the data quality of the remaining patient population was thereby increased. To our knowledge, this is one of the first non-interventional studies where quality assurance procedures were used to detect data falsification.

Reflecting current clinical experience, the microorganisms isolated most frequently were *S. pneumoniae* (including penicillin-resistant strains), *H. influenzae* (including β-lactamase producing strains), and atypical pathogens. Almost one-half of patients (41.4%) had received an antibiotic treatment in the previous 14 days for their current CAP episode before starting moxifloxacin therapy. The majority of these patients were switched to moxifloxacin because of a lack of efficacy with the previous antibiotic.

The primary objective of CAPRIVI was the distribution of the CRB-65 score at baseline. In this population, 87.5% of hospitalized patients had a CRB-65 score 0 or 1, while only 12.5% had a score 2 to 4. It had been predicted that a greater proportion of the patients would have a CRB-65 score of 2 or above, since a high score is a strong indicator for hospitalization. This study suggests that a large proportion of CAP patients are hospitalized in the participating countries who would be treated at home according to current international recommendations [5,40]. Potential explanations for this unexpected finding may include a high rate of failure of previous alternative antibiotic therapy (which occurred in 41.4% of patients), as well as differences in health care systems, such as a reluctance of primary care physicians to treat CAP patients in their homes, social circumstances that required greater rates of hospitalization, greater severity of underlying medical diseases in this study population, and the lower hospital costs in Eastern and South Eastern Europe versus Western Europe. The predictive power of the CRB-65 score for CAP-associated mortality appears to be supported in this study, as the mortality rate of hospitalized patients in the CAPRIVI study (<1%) was substantially lower than in hospitalized patients in the USA (10%–14%) and corresponds to the mortality rate in US patients treated in the community [54].

Moxifloxacin at a dose of 400 mg once daily for a mean of 10.0 days (range, 2.0–39.0 days) was a highly effective treatment in CAP patients. Improvements were reported in a range of diagnostic measures routinely used in clinical practice, including the patient’s overall condition, severity of infection, symptoms of dyspnea, cough, sputum, and thoracic pain, and rates of abnormal auscultation, core temperature, and chest radiography. In total, 96.7% of patients experienced an improvement, i.e., felt better during the study. Improvements occurred after a mean of 2.7 days, and over 94% of patients had experienced an improvement after 5 days; 93.2% of patients were reported cured of infection by the end of therapy. No differences in the efficacy of moxifloxacin were observed between patients who received moxifloxacin by intravenous administration alone or by sequential intravenous then oral administration. Also, no differences in the efficacy of moxifloxacin were observed between patients experiencing a range of disease severities at baseline.

In recent years, the susceptibility of typical pathogens to penicillins, macrolides, trimethoprim/sulfamethoxazole, and second-generation cephalosporins has decreased, so that multidrug-resistant strains causing CAP are of increasing clinical relevance [7,25,55]. It is notable that, despite this trend, moxifloxacin was effective in a large proportion of patients in this study.

Unlike in interventional trials, the moxifloxacin dosing regimen used in this non-interventional study was left to the discretion of the treating physician. It is interesting to note the high rate of physician compliance with the moxifloxacin dose recommended in the Summary of Product Characteristics (i.e., 400 mg daily). This suggests that the physicians considered the recommended dose of moxifloxacin to be highly effective, without need to adjust the dose, e.g., for body weight. The lack of need for dose adjustment has the advantages of easier dosing and a reduced risk of overdosing.

The cure rates of moxifloxacin reported in the CAPRIVI study are in agreement with previous controlled studies of patients with CAP (e.g, Finch et al. [23]; Torres et al. [30]; Hoeffken et al. [26]; Petitpretz et al. [27]). Ewig et al., in the observational CAPNETZ trial of CAP patients, reported high rates of survival and low rates of treatment failure for moxifloxacin relative to β-lactam monotherapy and β-lactam combination therapy, with a particular survival benefit for moxifloxacin in patients with high CRB-65 scores [56]. The rapid recovery from symptoms observed in CAPRIVI is a desirable characteristic for an effective treatment in patients with CAP. Other clinical studies have reported that moxifloxacin is associated with more rapid recovery from symptoms than other commonly used treatments [22].

The incidences of TEAEs and drug-related TEAEs in CAPRIVI were low and no deaths occurred in patients with TEAEs. The nature and the frequency of drug-related TEAEs (i.e., mainly gastrointestinal disorders) were consistent with the established safety profile of moxifloxacin. For most patients in the study, the TEAEs resolved during the course of treatment and were associated with low rates of treatment withdrawal (24 [0.9%] patients in total, including 19 [0.7%] patients with drug-related TEAEs).

Overall satisfaction with moxifloxacin treatment was high among both physicians and patients. Physicians rated the CAP symptoms “very much improved” or “much improved” in 95.3% of patients, while 94.1% of patients were “very much satisfied” or “much satisfied” with moxifloxacin therapy.

Limitations of the current study include the primary role of physician judgment in the decisions on patient selection and management; a lower than expected proportion of high-risk patients; and the absence of a control group to quantify the response to other antibacterial agents. A strength of CAPRIVI, as with other observational studies, is that it provides an accompaniment to randomized controlled trials by reflecting real-world practice in prescribing behavior.

## Conclusions

The efficacy and safety profiles of moxifloxacin characterized in this large observational study from Eastern and Central Europe and the Middle East confirm previous studies which report that moxifloxacin offers benefits in the treatment of inpatients with CAP. The high response rate in this study, which included patients with a range of disease severities, suggests that treatment with broader-spectrum drugs such as moxifloxacin is appropriate for patients with CAP who are managed in hospital.

## Abbreviations

CAP: Community acquired pneumonia; TEAEs: Treatment-emergent adverse events; SD: Standard deviation.

## Competing interests

IK, AB, IKT, LR, and LI declare that they have no competing interests. H-PM and TP are full-time employees of Bayer AG.

## Authors’ contributions

IK, AB, IKT, LR, and LI participated in data acquisition and data analysis and interpretation. H-PM and TP participated in study design and concepts, and data analysis and interpretation. All authors read and approved the final manuscript.

## Pre-publication history

The pre-publication history for this paper can be accessed here:

http://www.biomedcentral.com/1471-2466/14/105/prepub
